# Cerebrospinal fluid abnormalities in meningeosis neoplastica: a retrospective 12-year analysis

**DOI:** 10.1186/s12987-017-0057-2

**Published:** 2017-03-28

**Authors:** Marija Djukic, Ralf Trimmel, Ingelore Nagel, Annette Spreer, Peter Lange, Christine Stadelmann, Roland Nau

**Affiliations:** 1 Department of Geriatrics, Evangelisches Krankenhaus Göttingen-Weende, Göttingen, Germany; 2 Institute of Neuropathology, University Medical Center Göttingen (UMG), Robert-Koch-Strasse 40, 37075 Göttingen, Germany; 3 Department of Neurology, University Medical Center Göttingen (UMG), Göttingen, Germany; 4 Department of Neurology, University Medical Centre Mainz, Mainz, Germany

**Keywords:** Meningeosis neoplastica, Meningeosis carcinomatosa, Meningeosis lymphomatosa, Lactate, Carcinoembryonic antigen (CEA), CSF/serum albumin ratio

## Abstract

**Background:**

Meningeosis neoplastica is a diffuse metastatic spread of tumor cells in the subarachnoid space. Although first recognized in 1870, systematic investigations regarding cerebrospinal fluid (CSF) constituents in this condition are scarce.

**Methods:**

Routine CSF samples analyzed from 2001 to 2012 at the Laboratory of Clinical Neurochemistry, University of Göttingen, were re-evaluated. Patients, whose CSF contained malignant cells were included in this study.

**Results:**

Patients (n = 132, age 59.1 ± 29.1, 58% women) were identified, whose CSF contained malignant cells. The most frequent primary tumor was breast cancer (32.6%), followed by lung cancer (25.0%) and hematologic malignancies (21.2%). The most frequent clinical symptoms were affections of cranial nerves (41.7%), psychiatric abmormalities (32.6%) and radicular lesions of the lower extremities (20.5%). CSF cell counts ranged from 0 to 4692 cells/μl (median 4 cells/μl) and were elevated in 50%. The CSF-to-serum albumin ratio was abnormal in 69.4%. It ranged from 1.8 to 330 x 10^-3^ (median 17.5 x 10^-3^). Total CSF protein ranged from 166 to 15,840 mg/l (median 1012 mg/l). CSF lactate was elevated (>2.4 mmol/l) in 65.2% [3.6 mmol/l (1.3/15.6 mmol/l); median (minimum/maximum)]. In 50% of all patients CSF lactate was ≥3.5 mmol/l. The CSF cell counts correlated significantly with the CSF lactate levels and the CSF protein contents. In 56 of 118 CSF samples (47.5%) ferritin was elevated, and in 25 of 65 carcinoma patients (38.5%) an intrathecal production of carcinoembryonic antigen (CEA) was detected. Granulocytes were found in 52.7% of the CSF samples. The percentages of granulocytes and lymphocytes were higher in samples with an elevated cell count.

**Conclusion:**

In approximately 50% of CSF samples with meningeosis neoplastica the CSF cell count is not elevated. Diagnosis may be missed when only CSF samples with elevated cell counts are subjected to cytological analysis. CSF lactate and protein and the CSF-to-serum albumin ratio are frequently increased in meningeosis neoplastica. The differential diagnosis between meningeosis neoplastica and central nervous infections, in particular tuberculous or fungal meningitis, can be difficult.

## Background

Meningeosis neoplastica, the infiltration of the meninges and the subarachnoid space by malignant cells as a consequence of metastatic cancer, was first described by Karl Joseph Eberth as early as 1870 [1]. Meningeosis neoplastica is the generic term for all infiltrations of the meninges by malignancies including (1) Meningeosis carcinomatosa as the metastatic spread of a carcinoma to the meninges, (2) Meningeosis lymphomatosa with leptomeningeal involvement by hematologic malignancies and (3) dissemination to the meninges of primary tumors of the central nervous system, e.g. germinomas, medulloblastomas, primitive neuroectodermal tumors, ependymomas and malignant gliomas. Meningeosis carcinomatosa occurs in 3–8% of all cancer patients. Among solid tumors, the most frequent tumor types associated with meningeosis carcinomatosa are carcinomas of the lung and breast, and melanoma. Meningeosis lymphomatosa can be observed in approximately 5–15% of patients with hematologic malignancies. Meningeal involvement is most common with high-risk lymphomas and acute lymphocytic leukemia [2, 3]. Tumor cells migrate into the meninges either hematogeneously via small meningeal arteries and veins or by direct infiltration from the vicinity, i.e., from metastases or primary tumors in the skull, spinal cord or brain [4, 5]. After entry into the subarachnoid space or ventricles, malignant cells spread with the cerebrospinal fluid (CSF) along the whole CSF space. These cells frequently accumulate in regions with a reduced circulation velocity of the CSF, i.e., in the basal cisterns, the cauda equina or the hippocampal fissure [2].

Frequent clinical symptoms suggesting meningeosis neoplastica are headache, changes in mental status, difficulty in walking, nausea, vomiting, diplopia, lower motor weakness, limb paresthesia, back or neck pain, and radiculopathy [6]. Many antineoplastic drugs do not readily cross the blood–CSF and blood–brain barrier, but the doses of antineoplastic drugs necessary to produce effective CSF concentrations after direct injection into the CSF space are comparatively low (e.g., 10–15 mg for methotrexate, 40 mg for cytosine–arabinoside) [7]. For this reason, high antineoplastic drug concentrations in the CSF with low systemic toxicity can be reached by intrathecal chemotherapy. The magnetic resonance tomographic and CSF findings in meningeosis neoplastica can be confounded with infectious diseases of the CNS, particularly CNS tuberculosis and fungal meningoencephalitis.

An early diagnosis of meningeosis neoplastica, before persisting neurologic deficits have developed, permits earlier and potentially more effective treatment, thereby leading to a better quality of life in affected patients [6]. Since the indication for intrathecal chemotherapy relies on the detection of malignant cells in the CSF, all efforts must be undertaken to firmly establish the diagnosis. The present study aims at characterizing the CSF findings in a large group of patients with meningeosis neoplastica. Special emphasis was placed on the possible contribution of routine parameters for the differential diagnosis between meningeosis neoplastica and infectious or autoimmune diseases of the CNS.

## Methods

### Patients

The medical files including lumbar or ventricular CSF of patients with meningeosis neoplastica, who were treated between January 1, 2001, and December 31, 2012, with different clinical symptoms in the University Hospital Göttingen, in the Protestant Hospital Göttingen-Weende and other regional hospitals, were retrospectively analyzed. The inclusion criterion was the presence of tumor cells as assessed by morphological criteria. Clinical symptoms were assessed by review of the patients’ medical files. We also assessed the results, when cranial computer tomography (CCT) or magnetic resonance imaging (CMRI) was performed. The study was approved by the Ethics Committee of the Medical Faculty of the Georg-August University Göttingen, Germany.

### CSF analysis

After lysis of the erythrocytes, CSF cells were counted manually in a Fuchs-Rosenthal chamber. A cell count of ≤4/μl was considered normal. CSF cell differentiation was performed after cell sedimentation by means of a cytocentrifuge (Omnifuge 2.0 ORS, Thermo Scientific, Darmstadt, Germany) and May-Grünwald-Giemsa (MGG) staining (Merck, Darmstadt, Germany). Cytology was performed on MGG-stained cytospins. The appearance of tumor cells depended on the origin of the primary tumor. Tumor cells were in general larger than mononuclear hematopoietic cells. Furthermore, they frequently showed a marked increase in the nuclear/cytoplasmic ratio, basophilic cytoplasm, hyperchromasia, irregular nuclear membrane, pseudopodia and increased number of nucleoli. Especially in the case of epithelial cancer, tumor cells frequently lay in clusters and occasionally, in part atypical, mitotic figures were observed. Depending on the primary tumor, tumor cells occasionally showed evidence for mucus production or pigmentation. The CSF protein content was measured by nephelometry after precipitation with trichloroacetic acid in a Dosascat nephelometer (Dosatec, Gilching, Germany) using Gesamteiweiß UC Standard FS (DiaSys Diagnostic Systems, Holzheim, Germany) [8]. CSF and serum albumin, and immunoglobulins IgG, IgA and IgM were determined by nephelometry (BN ProSpec, Siemens Healthcare Diagnostics, Tarrytown, NY, USA), and the respective albumin and immunoglobulin CSF/serum quotients (IgG, IgA, IgM) were calculated and plotted in Reiber-Felgenhauer nomograms [9]. CSF oligoclonal IgG bands were detected by isoelectric focusing in polyacrylamide gels; for the comparison of CSF and serum bands samples were blotted at equalized IgG concentrations and, after immobilization on a nitrocellulose membrane, stained with anti-human IgG antibodies [9–11]. CSF lactate was determined enzymatically using the lactate oxidase reaction cleaving lactate into pyruvate and hydrogen peroxide. The resulting chinonimine concentration, which was proportional to the CSF lactate concentration, was quantified in an ELISA reader at 620 nm. CSF lactate concentrations ≤2.4 mmol/l were considered normal; CSF lactate concentrations ≥3.5 mmol/l are generally considered an indicator of CNS bacterial infection.

In addition to cytology, protein biomarkers were established especially for carcinomas invading the CNS and the leptomeninges [12]. Carcinoembryonic antigen (CEA), an oncofetal antigen and tumor marker particularly for gastrointestinal, breast or lung adenocarcinoma, was found to be secreted into the CSF of patients with meningeosis carcinomatosa [13]. Carcinoembryonic antigen (CEA) in CSF and serum was measured by enzyme immunoassay using antibody-coated beads and a CSF volume of 3 ml in order to increase the sensitivity of the assay [10, 14]. Since the molecular mass of CEA and IgA is approximately equal, intrathecal secretion of CEA was quantified by using the Reiber-Felgenhauer nomogram for the immunoglobulin IgA [14].

The CSF concentration of ferritin was measured by nephelometry (BN ProSpec, Siemens Healthcare Diagnostics, Tarrytown, NY, USA).

### Statistics

Statistical calculations were performed with GraphPad Prism software (GraphPad Software, La Jolla, USA). Data was described by means ± standard deviations (SD), when normally distributed. In the absence of normal distribution data was described by medians and interquartile ranges or as medians and ranges (minima/maxima). For univariate analyses, we used Mann–Whitney U test in the absence of normal distribution. The relation between CSF cell count and CSF lactate concentration and CSF protein was assessed by Spearman’s rank correlation coefficient, r_S_. p values ≤0.05 were considered statistically significant.

## Results

A group of 132 patients with meningeosis neoplastica [age 16–97 years (59.1 ± 29.1 years; mean ± SD), 77 women, 55 men)] was included in this study. In 128 patients lumbar CSF and in four patients ventricular CSF was studied.

**Fig. 1:**
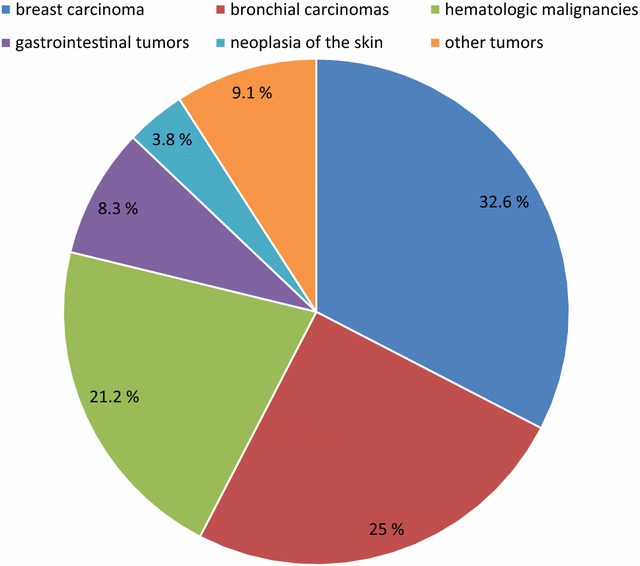
Pie chart of the frequency of various malignant diseases in the study population

Clinical symptoms of the patients studied are depicted in Table 1. Cranial nerve palsies were most frequent, followed by psychiatric abnormalities (mainly fluctuating level of consciousness and confusion) and radicular symptoms of the lower extremities and headache. In 32.6% breast carcinoma was the primary malignoma, followed by bronchial carcinomas (25.0%), hematologic malignancies (21.2%), gastrointestinal tumors (8.3%) and neoplasias of the skin (3.8%). All other malignancies including primary brain and spinal tumors accounted for 9.1% of the cases (Fig. 1).

**Table 1:** Clinical symptoms of the patients studied (n = 132) upon admission

Symptoms in the order of decreasing frequency	All patients n (%)
Cranial nerve palsy	55 (41.7%)
Psychiatric abnormalities	43 (32.6)
Radicular motor symptoms of the lower extremities	27 (20.5)
Headache	25 (18.9)
Hemiparesis	23 (17.4)
Nausea/vomiting	22 (16.7)
Radicular sensory symptoms of the lower extremities	21 (15.9)
Non-radicular sensory abnormalities	18 (13.6)
Para- or tetraparesis	17 (12.9)
Aphasia	14 (11.4)
Nuchal rigidity	12 (9.1)
Cognitive decline	11 (8.3)
Radicular motor symptoms of the upper extremities	11 (8.3)
Epileptic seizures	10 (7.6)
Radicular sensory symptoms of the upper extremities	5 (3.8)
Fever	2 (1.5)

Cranial nerve palsies were most frequent, followed by psychiatric abnormalities (mainly fluctuating level of consciousness and confusion)

97 patients with cytologically proven meningeosis neoplastica received cranial imaging [CCT (n = 19), CMRI (n = 24) or both (n = 54)] within a short time period before or after the diagnostic lumbar or ventricular CSF analysis. In 50 of these patients (51.5%), typical signs of meningeosis neoplastica were present on CCT or CMRI.

The results of CSF analysis in patients with meningeosis neoplastica are presented in Table 2. The CSF cell count (leukocytes and tumor cells cannot be distinguished reliably in the Fuchs-Rosenthal cytometer, and erythrocytes were lysed before counting) was elevated (>4 cells/μl) in the CSF samples of 66 patients and normal (≤4 cells/μl) in the other 66 patients. CSF pleocytosis ≥100 cells/µl was present in 18 patients (13.6%) , and four patients had a CSF pleocytosis ≥500 cells/μl. Malignant cells were detected in all CSF samples (inclusion criterion). Differentiation into lymphocytes, monocytes and granulocytes was carried out in the CSF of 55 patients (28 of them with normal cell count and 27 with pleocytosis).

**Table 2:** Cerebrospinal fluid analysis in patients with meningeosis neoplastica

Parameter	Normal findings n (%)	Abnormal findings n (%)	Median (minimum; maximum)
CSF cells (/µl) excluding erythrocytes	66 (50)	66 (50)	4 (0/4692)
CSF protein (mg/l)	28 (21.5)	102 (78.5)	1012 (166/15,840)
Q_Albumin_ × 10^−3^	37 (30.6)	84 (69.4)	17.5 (1.8/330)
CSF lactate (mmol/l)	46 (34.8)	86 (65.2)	3.6 (1.3/15.6)

In 50% of CSF samples with meningeosis neoplastica the CSF cell count was not elevated. The CSF-to-serum albumin ratio was abnormal in 69.4%, and total CSF protein was elevated (>450 mg/l) in 102 of 130 patients (78.5%). CSF lactate was elevated (>2.4 mmol/l) in 65.2%

In 32.6% breast cancer was the primary malignoma, followed by bronchial carcinomas (25.0%), hematologic malignancies (21.2%), gastrointestinal tumors (8.3%) and neoplasias of the skin (3.8%). All other malignancies including primary brain and spinal tumors accounted for 9.1% of the cases

Overall, lymphocytes were the most abundant cell population. Granulocytes were found in 52.7% of the CSF samples, in only two patients was the percentage of granulocytes >50%. Both of them had more than 500 cells/µl. In the CSF of patients with >4 cells/μl, lymphocytes and granulocytes were significantly more frequent than in the CSF with ≤4 cells/μl [lymphocytes 92% (72/94%) versus 77.5% (67/87.8% ), median (25th/75th quartile) (Mann-WhitneyU test: p < 0.01); granulocytes 19% (2/31%) versus 3.5% (0/8.3%) (Mann–Whitney U test: p = 0.013). Conversely, monocytes were less frequent in CSF samples with pleocytosis than in those with a normal cell count [8% (6/14%) versus 12% (10/20%), p < 0.01].

**Fig. 2:**
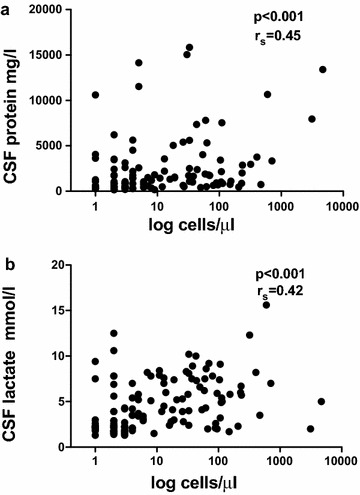
The CSF protein (n = 130) **a** and CSF lactate levels (n = 132) **b** correlated with the CSF cell counts (Spearman’s rank correlation coefficient r_S_ = 0.45, p < 0.001 for CSF protein and r_S_ = 0.42, p < 0.001 for lactate)

Total CSF protein was elevated (>450 mg/l) in 102 of 130 patients (78.5%). The CSF protein was positively correlated with the CSF cell count (n = 130, Spearman’s rank correlation coefficient r_S_ = 0.45, p < 0.001) (Fig. 2a). The CSF-to-serum albumin ratio was abnormal in 84 of 121 patients (69.4%). Intrathecal synthesis of IgG or IgA or IgM antibodies as assessed by the Reiber–Felgenhauer nomograms was detected in the CSF of 13 patients: four showed an isolated intrathecal synthesis of IgG, two synthesis of IgA, and nine synthesis of IgM. In two patients, nephelometry suggested synthesis of more than one class of antibodies (one patient: IgG plus IgM; one patient: IgG plus IgA). Nephelometry detected intrathecal immunoglobulin synthesis in 10 lymphoma and three carcinoma patients. By isoelectric focusing, intrathecal IgG synthesis was detected in the CSF of 25 of 62 patients (40.3%), i.e., isoelectric focusing was by far more sensitive than nephelometry in detecting intrathecal IgG synthesis.

Cerebrospinal fluid lactate was elevated (>2.4 mmol/l) in 65.2% [3.6 mmol/l (1.3/15.6 mmol/l); median (minimum/maximum)]. In 66 of 132 patients (50%), CSF lactate was strongly elevated (≥3.5 mmol/l). The CSF lactate concentration was correlated with the CSF cell count (n = 132, Spearman’s rank correlation coefficient r_S_ = 0.42, p < 0.001) (Fig. 2b).

Ferritin and CEA were not measured in all samples, but only upon request of the clinician in charge of the patient. In 56 of 118 CSF samples (47.5%) ferritin was elevated, and in 25 of 81 cases (30.9%) an intrathecal production of CEA was detected. No intrathecal CEA production was detected in lymphoma patients. Of the 65 patients with meningeosis carcinomatosa, 25 patients (38.5%) had intrathecal CEA synthesis.

## Discussion

Clinical signs and symptoms of the patients included in this study were very similar to those reported by others [6]. CSF analysis is the key laboratory investigation to diagnose/confirm meningeosis neoplastica. If this diagnosis is suspected, repeated analyses of CSF may be necessary to prove the presence of malignant cells in the CSF, as this is the diagnostic cornerstone of meningeosis neoplastica. Furthermore, the detection of malignant cells in CSF is the prerequisite for the start of intrathecal therapy, and one criterion for successful treatment is the disappearance of tumor cells. In our study the presence of malignant cells in CSF was the inclusion criterion, i.e., all CSF samples evaluated contained malignant cells. Surprisingly, 50% of the CSF samples included had normal cell counts. In a previous study, 32% of patients with cytologically-proven meningeosis neoplastica had normal CSF cell counts [15]. These studies illustrate that clinicians cannot rely on a normal CSF cell count to exclude meningeosis neoplastica, and cytological examination of CSF should always be included in the routine work-up of CSF analysis. Careful cytopathologic analysis is necessary in all CSF samples, when infiltration of the CSF space by malignant cells is suspected.

When the CSF cell count is elevated and malignant cells are not present or are overlooked, differentiation between meningeosis neoplastica and infections or autoimmune diseases affecting the CSF space can be difficult. In particular, tuberculous meningitis can be confounded with meningeosis neoplastica, because the clinical symptoms and CSF abnormalities can be similar. Moreover, many patients suffering from malignant diseases are immunosuppressed either by chemotherapy or the underlying disease. They, therefore, are prone to develop CNS infections with *M. tuberculosis*, fungi and other opportunistic pathogens, which also can cause CSF alterations similar to those observed in meningeosis neoplastica.

Elevated CSF lactate is not specific for certain diseases. Meningeosis neoplastica can cause a CSF lactate elevation [16], but a concentration ≥3.5 mmol/l is considered an indicator of bacterial or fungal meningoencephalitis [11]. Most of the CSF lactate originates from host cells, in particular, leukocytes and neurons. Even in bacterial meningitis, ≥95% of the CSF lactate originates from anaerobic glycolysis of host cells and not from bacteria in the CSF [17]. In the present study, CSF lactate was correlated with the CSF cell count. In a recent receiver operating characteristic (ROC) curve analysis, CSF lactate had the highest accuracy for discriminating bacterial from viral meningitis with a cutoff set at 3.5 mmol/l [18]. Using a cutoff of 3.5 mmol/l, CSF lactate was also very useful to discriminate between cryptococcal or tuberculous meningitis and HIV chronic meningitis [19]. In acute Lyme neuroborreliosis, an atypical bacterial CNS infection with low pathogen concentrations in CSF and symptoms, which may resemble meningeosis carcinomatosa (in particular, radicular lesions and cranial nerve involvement), only 5 of 118 patients (4%) had a CSF lactate ≥3.5 mmol/l, and the mean CSF lactate level was not elevated [20]. This implies that CSF lactate concentrations ≥3.5 mmol/l can help in discriminating meningeosis neoplastica from viral meningitis, neuroborreliosis, mitochondriopathy and many other conditions, but not from neurotuberculosis and fungal meningoencephalitis.

Meningeosis neoplastica frequently is associated with an inflammatory response. The presence of intrathecal IgG, IgA and IgM synthesis as well as the high proportion of lymphocytes in the CSF of patients with meningeosis neoplastica indicate a strong involvement of the adaptive immune system. As in neuroborreliosis, isoelectric focusing was more sensitive for detecting an intrathecal IgG synthesis than the Reiber–Felgenhauer nomogram [20]. The presence of granulocytes in the CSF in neoplastic meningeosis suggests a contribution of the innate immune system to the inflammatory reaction. In tuberculous meningitis which also is characterized by a strong elevation of CSF lactate and total protein and a moderate elevation of cell counts (10–500 cells/μl), neutrophil predominance (>50%) [21] was a strong predictor of tuberculous meningitis with a sensitivity of 54% and a specificity of 98% [22]. Since only two of the 55 CSF samples in our study, where differential cell counts were carried out, contained >50% granulocytes, neutrophilic predominance in CSF is an argument against the diagnosis of meningeosis neoplastica. However, in nearly 50% of patients with tuberculous meningitis, neutrophil predominance is absent, and the differentiation between these two diseases relies on bacteriologic exams (Ziehl-Neelsen stain, culture, polymerase chain reaction) and on the cytological identification of tumor cells.

The CSF protein concentration correlated positively with the CSF cell count. One explanation for this is an abnormal blood–CSF barrier function with increased permeability for leukocytes and serum-derived proteins. In particular, the leptomeningeal structures and the epithelial cells of the choroid plexus can be infiltrated by neoplastic cells, resulting in an increased permeability for serum compounds [23]. Another explanation for this correlation is a decreased CSF flow rate due to the obstruction of the sites of CSF absorption, especially arachnoid granulations and the perineural spaces of the cranial and spinal nerves by malignant cells or leukocytes. As a consequence of the CSF flow reduction, the protein gradient between serum and CSF can decrease [24].

Ferritin CSF concentrations were elevated in 56 of 118 patients (47.5%). CSF ferritin concentrations are elevated after intracranial bleeding and in neoplastic and meningitic CNS diseases, but this parameter is neither specific nor sensitive. An elevated CSF ferritin reliably, but non-specifically indicates severe CNS disease [25].

Carcinoembryonic antigen was measured in 81 patients. In patients with metastatic CEA-producing tumors, diffusion of CEA across the blood–CSF and blood–brain barrier cannot be neglected. The CSF concentrations depend on the respective serum concentration, and absolute cut-off values are not appropriate. Because of the similar molecular mass of CEA and IgA, the Reiber–Felgenhauer nomogram for IgA can be used to detect intrathecal CEA synthesis [10, 14, 26]. In 25 patients of 65 carcinomas cases (38.5%) the Reiber–Felgenhauer nomogram indicated intrathecal CEA synthesis. Using the IgA nomogram, the detection of intrathecal CEA is considered a very specific, but not very sensitive indicator of meningeosis carcinomatosa or cerebral metastases. A normal or undetectable CSF-to-serum ratio of CEA does not exclude infiltration of CNS compartments by carcinoma cells, lymphomas and primary brain tumors do not produce CEA [14]. Another method to detect intracranial synthesis of CEA compares the CSF-to-serum ratio of CEA with the CSF-to-serum albumin ratio and assumes intrathecal synthesis when the CSF-to-serum ratio of CEA is greater than the CSF-to-serum albumin ratio determined on the same day. Since the molecular mass of CEA is larger than albumin, this method is less sensitive than the use of the IgA nomogram [10]. We doubt that CEA CSF concentrations without knowledge of the corresponding serum levels are of value for the detection of meningeosis carcinomatosa. Others, however, have reported that the combination of CSF concentrations of CEA, neuron specific enolase (NSE) and cytokeratin 19 fragments (CYFRA21-1) using cut-off values without consideration of the respective serum levels appears to be useful for the detection of meningeal carcinomatosis of lung cancer [27].

## Conclusions

The two main findings of this study are: (1) In half of the CSF samples with meningeosis neoplastica the CSF cell count is not elevated. Therfore, diagnosis can be missed when only CSF samples with elevated cell counts are subjected to cytological analysis. (2) CSF lactate and protein and the CSF-to-serum albumin ratio are frequently increased in meningeosis neoplastica. The differential diagnosis between meningeosis neoplastica and central nervous infections, in particular tuberculous or fungal meningitis, can be difficult. For the detection or exclusion of malignant cells in the CSF, cytology performed by an experienced pathologist is necessary.

## Abbreviations

CEA: carcinoembryonic antigen
CCT: cranial computer tomography
CMRI: cranial magnetic resonance imaging
CSF: cerebrospinal fluid
CYFRA21-1: cytokeratin 19 fragments
MGG: May-Grünwald-Giemsa
NSE: neuron specific enolaseMGG May-Grünwald-Giemsa

## References

[1] Eberth CJ (1870). Zur Entwicklung des Epitheliomas (Cholesteatomas) der Pia und der Lunge. Virchow’s Arch.

[2] Grossman SA, Krabak MJ (1999). Leptomeningeal carcinomatosis. Cancer Treat Rev.

[3] Kaplan JG, DeSouza TG, Farkash A, Shafran B, Pack D, Rehman F, Fuks J, Portenoy R (1990). Leptomeningeal metastases: comparison of clinical features and laboratory data of solid tumors, lymphomas and leukemias. J Neurooncol.

[4] Rosen ST, Aisner J, Makuch RW, Matthews MJ, Ihde DC, Whitacre M, Glatstein EJ, Wiernik PH, Lichter AS, Bunn PA (1982). Carcinomatous leptomeningitis in small cell lung cancer: a clinicopathologic review of the national cancer institute experience. Medicine (Baltimore).

[5] http://www.dgn.org/leitlinien/2979-ll-77-metastasen-und-meningeos-neoplastica Accessed 15 Dec 2016.

[6] Le Rhun E, Taillibert S, Chamberlain MC (2013). Carcinomatous meningitis: leptomeningeal metastases in solid tumors. Surg Neurol Int.

[7] Morikawa N, Mori T, Kawashima H, Fujiki M, Abe T, Kaku T, Konisi Y, Takeyama M, Hori S (1998). Pharmacokinetics of nimustine, methotrexate, and cytosine arabinoside during cerebrospinal fluid perfusion chemotherapy in patients with disseminated brain tumors. Eur J Clin Pharmacol.

[8] Reiber H (1980). Eine schnelle und einfache nephelometrische Bestimmungsmethode für Protein im Liquor cerebrospinalis. J Clin Chem Clin Biochem.

[9] Reiber H, Felgenhauer K (1987). Protein transfer at the blood cerebrospinal fluid barrier and the quantitation of the humoral immune response within the central nervous system. Clin Chim Acta.

[10] Wick M Ausgewählte Methoden der Liquordiagnostik und klinichen Neurochemie, 2004. https://www.uke.de/extern/dgln/pdf/Methodenkatalog.pdf. Accessed 12 Dec 2016

[11] Wildemann B, Oschmann P, Reiber H (2006). Neurologische Labordiagnostik.

[12] Weston CL, Glantz MJ, Connor JR (2011). Detection of cancer cells in the cerebrospinal fluid: current methods and future directions. Fluids Barriers CNS.

[13] Jacobi C, Reiber H, Felgenhauer K (1986). The clinical relevance of locally produced carcinoembryonic antigen in cerebrospinal fluid. J Neurol.

[14] Petereit HF, Sindern E, Wick M (2007). Leitlinien der Liquordiagnostik und Methodenkatalog der Deutschen Gesellschaft für Liquordiagnostik und Klinische Neurochemie.

[15] Liu J, Jia H, Yang Y, Dai W, Su X, Zhao G (2009). Cerebrospinal fluid cytology and clinical analysis of 34 cases with leptomeningeal carcinomatosis. J Int Med Res.

[16] Hornig CR, Busse O, Kaps M (1983). Importance of cerebrospinal fluid lactate determination in neurological diseases. Klin Wochenschr.

[17] Wellmer A, Prange J, Gerber J, Zysk G, Lange P, Michel U, Eiffert H, Nau R (2001). d- and l-lactate in rabbit and human bacterial meningitis. Scand J Infect Dis.

[18] Giulieri S, Chapuis-Taillard C, Jaton K, Cometta A, Chuard C, Hugli O, Du Pasquier R, Bille J, Meylan P, Manuel O, Marchetti O (2015). CSF lactate for accurate diagnosis of community-acquired bacterial meningitis. Eur J Clin Microbiol Infect Dis.

[19] de Almeida SM, Boritza K, Cogo LL, Pessa L, França J, Rota I, Muro M, Ribeiro C, Raboni SM, Vidal LR, Nogueira MB, Ellis R (2011). Quantification of cerebrospinal fluid lactic acid in the differential diagnosis between HIV chronic meningitis and opportunistic meningitis. Clin Chem Lab Med.

[20] Djukic M, Schmidt-Samoa C, Lange P, Spreer A, Neubieser K, Eiffert H, Nau R, Schmidt H (2012). Cerebrospinal fluid findings in adults with acute Lyme neuroborreliosis. J Neurol.

[21] Thwaites GE, Chau TT, Stepniewska K, Phu NH, Chuong LV, Sinh DX, White NJ, Parry CM, Farrar JJ (2002). Diagnosis of adult tuberculous meningitis by use of clinical and laboratory features. Lancet.

[22] Zou Y, He J, Guo L, Bu H, Liu Y (2015). Prediction of cerebrospinal fluid parameters for tuberculous meningitis. Diagn Cytopathol.

[23] Schumacher M, Orszagh M (1998). Imaging techniques in neoplastic meningiosis. J Neurooncol.

[24] Reiber H (1994). Flow rate of cerebrospinal fluid (CSF)–a concept common to normal blood–CSF barrier function and to dysfunction in neurological diseases. J Neurol Sci.

[25] Kolodziej MA, Proemmel P, Quint K, Strik HM (2014). Cerebrospinal fluid ferritin—unspecific and unsuitable for disease monitoring. Neurol Neurochir Pol.

[26] Reiber H (2016). Cerebrospinal fluid data compilation and knowledge-based interpretation of bacterial, viral, parasitic, oncological, chronic inflammatory and demyelinating diseases. Diagnostic patterns not to be missed in neurology and psychiatry. Arq Neuropsiquiatr.

[27] Wang P, Piao Y, Zhang X, Li W, Hao X (2013). The concentration of CYFRA 21-1, NSE and CEA in cerebro-spinal fluid can be useful indicators for diagnosis of meningeal carcinomatosis of lung cancer. Cancer Biomark.

